# Opinion: A serious issue with the standardization of the adenovirus-based COVID-19 vaccines?

**DOI:** 10.1007/s00204-021-03126-9

**Published:** 2021-08-06

**Authors:** Michael Arand

**Affiliations:** Institute of Pharmacology and Toxicology, University of Zurich, Zurich, Switzerland

Dear Editor,

Since more than a year our world is in the grip of the COVID-19 pandemic. The necessary measures to reduce the incidence of infection have an enormous impact on our daily life. The way out appears to be an efficient vaccination campaign.

With unprecedented speed an impressive array of different vaccines has been developed and tested during the last year. These vaccines are based on different technological platforms, but converge in the strategy to elicit an immune response against the viral spike protein from the viral envelope that interacts with the angiotensin converting enzyme 2 (ACE2) protein on the surface of host cells to initiate their infection (Forni et al. 2021).

Classical approaches for vaccination, like the application of glutaraldehyde-inactivated viruses or purified recombinant spike protein, have been used with moderate success. However, some of the more innovative approaches that aim to reprogram body cells to produce the S1 protein for subsequent presentation to the immune system have turned out to be much more successful. Of these, the direct injection of spike protein-coding mRNA formulated as lipid nanoparticles to ensure sufficient stability and efficient uptake by host cells has demonstrated enormous efficacy. Comirnaty (BioNTech/Pfizer) and Spikevax (Moderna), the two mRNA-based vaccines that have obtained full market authorization in Switzerland and emergency authorization in many other countries, have both impressed with an efficacy of around 95% in preventing the infection (Baden et al. 2021; Polack et al. 2020). Likewise, the adenovirus-based vaccine Sputnik V (Gamaleya Research Institute) is reported to afford > 90% protection from infection (Logunov et al. 2020). Somewhat more disappointing were the reports for Vaxzevria (AstraZeneca), the Chimpanzee adenovirus-based vaccine meanwhile widely in use, with a reported protection efficacy between 60 and 90%, depending on the study (Voysey et al. 2021). Surprisingly, the counterintuitive observation was made that in a cohort that received a lower dose for the priming injection, a higher efficacy was obtained. What may be the reason for such an unexpected result? One explanation that has been put forward is a potential immunity against the vector, arising after the first immunization, that may simply be less pronounced with a lower initial dose. In turn, the booster injection may be more efficacious if the first dose was low. Alternatively, it could just be a false-positive result, a statistical outlier. However, an—in my personal view—very likely explanation has so far been overlooked. I will elaborate on that in the following.

A close look at the dosing regimen in the Vaxzevria clinical trials reveals that it is based on the number of viral particles: the standard dose applied per shot is 5 × 10^10^ particles, as determined by either quantification of viral protein or viral genomes (Voysey et al. 2021). However, the production of infectious adenovirus particles in HEK293 cells, the procedure widely applied for virus production, including the production of Vaxzevria, is a process that is difficult to standardize with respect to the final yield. Unfortunately, the percentage of viral particles that are actually infectious, i.e., those that can elicit an immune response against the recombinant protein they code for, can vary by orders of magnitudes. Tatsis and colleagues have reported that in their hands, several different Chimpanzee adenovirus-derived constructs showed batch-to-batch variations in the ratio of viral particles to infectious units by more than two orders of magnitudes (Tatsis et al. 2006). Thus, the particle (or genome) number is no adequate parameter for the estimation of the active principle, the infectious units, inside of an adenoviral preparation.

**Fig. 1:**
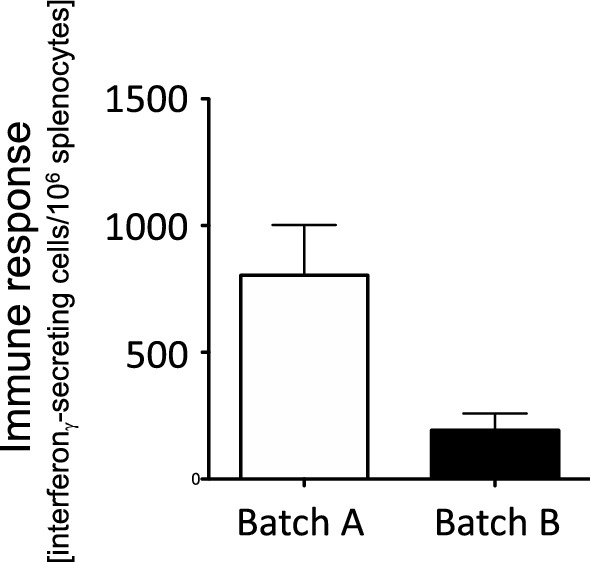
Immune response to an adenoviral vaccine, dosing based on viral particles. Immune responses were measured after intramuscular injection of 10^7^ adenoviral particles in Balb/c mice using two different batches of the same adenoviral construct. Batch A had a viral particle-to-infectious units ratio of 6.67, batch B had a viral particle-to-infectious units ratio of 47.88. Four mice were tested per batch. Data are given as mean ± SEM. The difference in the immune responses to the two batches was statistically significant (*p* = 0.013 in a one-sided students *T*-test). Data are taken from Dicks et al. (2012)

**Fig. 2:**
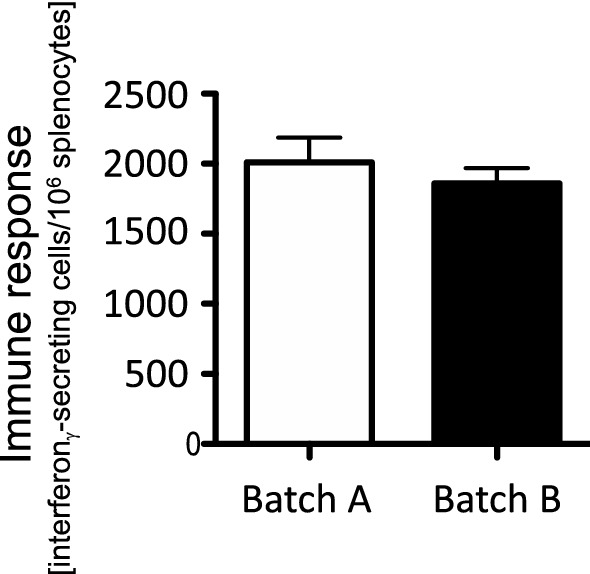
Immune response to an adenoviral vaccine, dosing based on infectious units. The same experimental paradigm and virus preparations as in Fig. 1 were used, with the single exception that the doses applied were 10^7^ infectious units, rather than viral particles. The difference in the immune responses to the two batches were not statistically significant (*p* = 0.250 in a one-sided students *T*-test). Data are taken from Dicks et al. (2012)

**Fig. 3:**
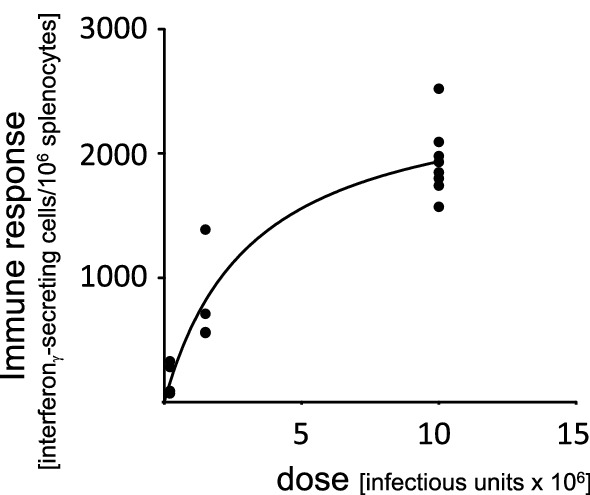
Dose response to an adenoviral vaccine, based on infectious units. Data from Fig. 1 and 2, for the sake of simplicity fitted to Michaelis–Menten equation (*r*^2^ = 0.892)

**Fig. 4:**
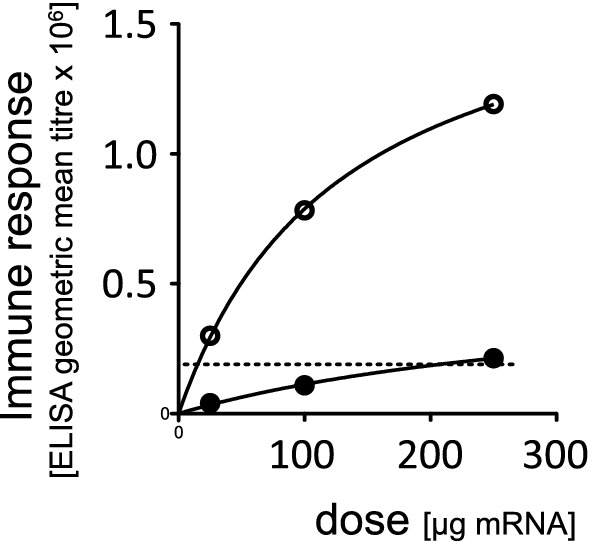
Dose response to an mRNA vaccine in a human clinical trial. Antibody titer in human blood sera as measured by an ELISA using the P2-variant of the spike protein (forced open conformation) as the antigen after immunization of healthy human individuals (*n* = 15). Immune responses were measured 4 weeks after the first (closed circles) or second (open circles) immunization. The dotted line indicates the geometric mean of data obtained from sera of COVID-19 patients after recovery. The data are taken from the phase I clinical trial of Spikevax (Jackson et al. 2020). Fitting the curves to the Michaelis–Menten equation resulted in r2 of 0.996 and 0.999 for the first and second immunization, respectively

In fact, when the backbone of the adenoviral vector finally used in Vaxzevria was developed, the authors paid attention to this fact and analyzed the relation of immune response to the viral dose, either defined by particle number or by infectious units (Dicks et al. 2012). When testing two different patches that differed substantially in their particle-to-infectious-units ratio (6.7 versus 48), they observed that dosing by viral particles produced a significantly different immune response in favor of the batch enriched in infectious units (Fig. 1), while dosing by infectious units produced an almost equal response for both batches (Fig. 2). Further analysis of their data reveals a clear dose–response when the immune response is plotted against the number of infectious units (Fig. 3). This substantiates that the active principle inside the preparation is the infectious virus and that its quantity is the relevant parameter for a proper dosing. That the dose–response is an important issue in human vaccination has nicely been demonstrated in the clinical phase I trial of Spikevax (Fig. 4), indicating that lower doses lead to substantially lower immune responses (Jackson et al. 2020).

Coming back to Vaxzevria and the apparent discrepancy between dosing schemes and efficacy, a likely explanation in view of the above facts is that batch differences in the ratio of viral particles to infectious units are the reason for the unexpected outcome, and that the improper dose adjustment to viral particles/genomes produces variable immunization results.

Why then has viral particle number been chosen as the basis for the dosing? Looking closer at other adenoviral vaccines, for instance Sputnik V and the Janssen COVID-19 vaccine (Logunov et al. 2020), their dosing is also based on viral particles. The reason for this collective—in my view inappropriate—adjustment is found in the legal framework: the European Pharmacopoeia states in its chapter 5.14 *Gene transfer medicinal products for human use* at the end of the passage specifying the requirements for *Adenovirus vectors for human use* under the heading *Labelling*: “The label states: … the recommended human dose, expressed in vector particle concentration; …”. This, in my personal opinion, is a highly unfortunate mistake. It appears that also the vaccine producing companies have meanwhile realized that dosing according to viral particles is not appropriate, and at least in the EU, the dosing is now given in infectious units, at least for Vaxzevria and the Janssen adenovirus-based vaccine, stating that at least 2.5 × 10^8^ (Vaxzevria (EMA_product_information_Vaxzevria 2021)) or 8.92 log_10_ (= 8.3 × 10^8^; Janssen vaccine (EMA_product_information_Janssen_vaccine 2021)) infectious units must be applied per dose. However, this still equals to a maximum ratio of viral particles to infectious units of 200 (Vaxzevria) or 60 (Janssen vaccine). Unfortunately, the clinical trials of both companies were based on viral particle number, 5 × 10^10^ per dose for both vaccines, without specifying the exact particle-to-infectious units ratio. Thus, we have no precise idea of the efficacy of the now defined minimal doses, and the content of the active principle of these vaccines may vary by a factor of 40 (Vaxzevria) or 12 (Janssen vaccine) if we take 5 as the lowest achievable ratio of viral particles to infectious units (reasonable estimate based on the data of Tatsis et al. (2006) and Dicks et al. (2012)). In my view, this is not acceptable. We would never tolerate a similar variability in the potency of ordinary medications, such as antibiotics, and we should aim for the same standards in the vaccines that we develop to save the world from the grip of the COVID pandemic. I therefore propose to change the wording in the chapter 5.14 of the European Pharmacopoeia to “…-the recommended dose, expressed in infectious units; …”. Furthermore, it might be informative to reanalyze the clinical trials and/or the postmarketing data of the adenovirus-based vaccines to gain a better understanding of the actual correlation between infectious units-based dosing and protection efficacy. I would hope to see that the achievable efficacy may well exceed the 90% if dosing is done properly, rendering adenoviral COVID vaccines valuable contributors to the defeat of the pandemic.
